# Expression of CD147 on monocytes/macrophages in rheumatoid arthritis: its potential role in monocyte accumulation and matrix metalloproteinase production

**DOI:** 10.1186/ar1778

**Published:** 2005-06-23

**Authors:** Ping Zhu, Jin Ding, Jun Zhou, Wei-Jia Dong, Chun-Mei Fan, Zhi-Nan Chen

**Affiliations:** 1 Department of Clinical Immunology, Xijing Hospital, Fourth Military Medical University, Xi'an, Shaanxi Province, China; 2 Department of Cell Biology, Fourth Military Medical University, Xi'an, Shaanxi Province, China

## Abstract

Monocytes/macrophages play an important role in rheumatoid arthritis (RA) pathogenesis. They can activate fibroblasts through many molecules, including IL-1 and tumor necrosis factor-alpha, but there have been very few reports on the role of CD147 in RA. In our study, the results of flow cytometry reveal that the mean fluorescence intensity (MFI) of CD147 expression on CD14+ monocytes of peripheral blood from RA patients was higher than that in normal control and ankylosing spondylitis (AS) patients. The MFI of CD147 expression on the CD14+ monocytes in RA synovial fluid was higher than that in RA peripheral blood. Immunohistochemical staining shows that CD147 expression in RA synovium correlated with matrix metalloproteinase (MMP)-1 expression. A double immunofluorescent assay shows that CD147 was expressed on CD68+ cells in RA synovium. The potential role of CD147 in cyclophilin A (CyPA)-mediated cell migration was studied using a chemotaxis assay *in vitro *and it was found that the addition of anti-CD147 antibody or a CD147 antagonistic peptide significantly decreased the chemotactic index of the mononuclear cells. The role of CD147 in MMP production and cell invasion *in vitro *were studied through the co-culture of human CD14+ monocytes or monocytic line THP-1 cells and human fibroblasts, as well as by gel zymography and an invasion assay. Significantly elevated release and activation of MMP-9 and/or MMP-2 were seen in the co-culture of human monocytes/THP-1 cells and fibroblasts compared with cultures of the cells alone. An increased number of cells invading through the filters in the invasion assays was also observed in the co-cultured cells. The addition of CD147 antagonistic peptide had some inhibitory effect, not only on MMP production but also on cell invasion in the co-culture. Our study demonstrates that the increased expression of CD147 on monocytes/macrophages in RA may be responsible for elevated MMP secretion, cell invasion and CyPA-mediated cell migration into the joints, all of which may contribute to the cartilage and bone destruction of RA. These findings, together with a better understanding of CD147, CyPA and RA, will help in the development of innovative therapeutic interventions for RA.

## Introduction

Monocytes/macrophages are known to play an important role in the pathogenesis of rheumatoid arthritis (RA). The number of monocytes/macrophages infiltrating into the rheumatoid synovium correlates with the extent of the inflammation in synovial tissues [1]. At the cartilage-pannus junction, macrophages, together with fibroblasts and endothelial cells, are important sources of matrix metalloproteinases (MMPs), which have been demonstrated to be involved in the process of cartilage and subchondral bone degradation [2,3]. The potential of macrophages to degrade cartilage matrix components may be modest, however, compared with that of synovial fibroblasts, which are thought to be possibly one of the principle cells involved in effecting the destructive response [4,5]. Thus, rather than the primary effector of tissue destruction, macrophages may act as an amplifier of the pathogenetic cascade, especially via activation of fibroblasts by molecules such as IL-1 and tumor necrosis factor (TNF)-alpha. Other molecules, such as CD147, also participate in this process and may play important roles in RA pathogenesis, but very few reports have been presented on their precise functions.

CD147 (also known as extracellular MMP inducer (EMMPRIN), basigin, tumor cell-derived collagenase stimulatory factor, human leukocyte activation-associated M6 antigen, or HAb18G) is a highly glycosylated immunoglobulin superfamily transmembrane protein [6,7]. It was initially identified on the surface of human cancer cells and has been proven to stimulate the adjacent stromal cells to produce several MMPs, including MMP-1, MMP-2, MMP-3, membrane type 1 MMP (MT1-MMP) and MT2-MMP [8-10]. Cellular expression analysis using the monoclonal antibodies from an international workshop on HLA indicates that CD147 is broadly expressed on haemopoietic and non-haemopoietic cell lines [11]. The CD147 expressed by monocytes/macrophages may similarly induce MMP production by fibroblasts and play an essential role in articular cartilage lesion development in RA. The expression of CD147 is upregulated in the rheumatoid arthritis synovial membrane and correlates with MMP-1, MMP-2, and MMP-3 upregulation [12,13]. There has been to date, however, no study reported on the expression of CD147 on monocytes/macrophages of synovial fluid and macrophage-like synoviocytes in RA.

The study reported here was designed to investigate the expression of CD147 on monocytes/macrophages of peripheral blood, synovial fluid and synovium in RA and to explore the possible functions of CD147 in the pathogenesis of RA. We found that CD147 was highly expressed on the monocytes of peripheral blood and synovial fluid in RA, and also that CD147 was expressed on CD68+ cells in RA synovium. Our *in vitro *functional assays of a co-culture of human monocytes or THP-1 cells and fibroblasts reveal a significantly elevated production of MMP-9 and/or MMP-2 and a significant increase in the number of cells invading through the Matrigel layer and filter compared with those in the respective cultures of these cells alone. CD147 antagonistic peptide had some inhibitory effect on MMP production and cell invasion in the co-culture. In the cyclophilin A (CyPA)-mediated cell migration assays, the addition of anti-CD147 antibody or CD147 antagonistic peptide significantly decreased the chemotactic index of the peripheral blood mononuclear cells.

## Materials and methods

### Patients

Samples of peripheral blood and synovial fluid were obtained from 15 patients with active RA. Joint synovium specimens were obtained from 12 patients with RA undergoing joint replacement surgery either at an affiliated hospital of Beijing University in Beijing or at Xijing Hospital in Xi'an. All the RA patients met the 1987 revised diagnostic criteria of the American College of Rheumatology [14]. The mean age of the patients was 56 years (range 28 to 74) and the mean disease duration was 9 years. The 15 patients with active RA from whom samples were obtained had received no treatment or were treated only with nonsteroidal anti-inflammatory drugs. Their mean erythrocyte sedimentation rate was 52 ± 28 mm/h and the C-reactive protein was 30 ± 28 mg/l. Samples used as control were obtained from another 15 patients with ankylosing spondylitis (AS) who met the 1984 modified New York criteria [15]. The mean age of the patients was 35 years (range 14 to 49) and the mean disease duration was 4 years. Joint specimens were obtained as control from five patients with osteoarthritis (OA) and three patients with AS. The normal control samples of peripheral blood were taken from 15 healthy human donor volunteers, with no significant age or sex differences compared to the RA patients. Ethics approval was granted for this study and all the subjects provided their informed consent.

### Flow cytometry analysis

Mononuclear cells from heparinized synovial fluid of RA patients were incubated with hyalidase (Sigma, Saint Louris, Missouri, USA) at 37°C for 30 minutes before being isolated using the Ficoll-Hypaque (Sigma) gradient centrifugation method. Peripheral blood cells were activated for 2 h using 50 u/ml IFN-γ (Sigma, Saint Louis, Missouri, USA). The concentration and incubation time were optimized by pre-experiment. According to the manufacturer's instructions, the whole blood cells or separated synovial fluid cells were labeled with FITC-conjugated anti-CD147 monoclonal antibody (or FITC-conjugated Mouse IgG1 for the control) (BD Pharmingen, San Diego, CA, USA) and PerCP-conjugated anti-CD14 monoclonal antibody (Becton-Dickinson, San Jose, CA, USA). The red cells in whole blood were lysed with a lysis reagent (Becton-Dickinson). Cells were analyzed with FACS Calibur flow cytometry (Becton-Dickinson). CD14+ cells were gated and 5000 events were measured. Data were processed using the Cell Quest software (Becton-Dickinson).

### Immunohistochemistry staining of synovium

Immunohistochemistry staining of the synoviums from 12 RA patients and controls (5 OA, 3 AS) was performed using a streptavidin/peroxides (SP) kit (Zymed, San Francisco, CA, USA) according to the manufacturer's instructions. The monoclonal antibodies used were anti-CD147 mab (Becton-Dickinson), anti-MMP-1 mab (NeoMarkers, Fremont, California, USA), and anti-TIMP-1 mab (NeoMarkers). Sections were reacted in turn with biotin labeled goat-anti-mouse IgG, horseradish peroxidase (HRP) labeled streptavidin avidin, and diaminobenzidine (DAB) (Zymed) before they were restained with haematoxylin for visulization of nuclei. For negative controls, primary antibodies were substituted by PBS instead of CD147 or MMP antibodies. In the positive section the cell membrane and/or cytoplasm were clear brown-yellowish in color.

### Laser scanning confocal microscope analysis of synovium

After fixation, the frozen-sections of synovial tissue were incubated first with rabbit anti-human CD147 polyclonal antibody (Zymed) and mouse anti-human CD68 monoclonal antibody (Serotec, Oxford, UK) and then with FITC-labeled goat anti-mouse IgG (Sigma) and CY3 labeled goat anti-rabbit IgG (Sigma). The sections were washed, mounted and then analyzed and photographed with an Olympus FV300 laser scanning confocal microscope (LSCM; Olympus FV300, Tokyo, Japan). Five hundred cells were counted in every section and distinct red, green or yellow fluorescence were observed in the membrane or cytoplasm of positive cells.

### Chemotaxis assay

The mononuclear cells were obtained from heparinized venous blood by the Ficoll-Hypaque (Sigma) gradient centrifugation method. The chemotaxis of CyPA was assessed in a 48-well modified Boyden chamber (Neuro Probe, Gaithersburg, Maryland, USA) with the two compartments separated by a polyvinylpyrrolidone-free polycarbonate filter with a 5 mm pore size (Neuro Probe). The mononuclear cells (1 × 10^6 ^cells/ml) in serum-free RPMI-1640 supplemented with 2% BSA were added to the compartment above the filter, and chemoattractants and negative controls (serum-free RPMI-1640 supplemented with 2% BSA) diluted in the same medium were put below the filters. The chambers were incubated at 37°C and 5% CO_2 _for 90 minutes before the filters were recovered, fixed and stained with Giemsa (Sigma, Saint Louis, Missouri, USA) reagent. The number of cells appearing on the lower face of the filter was recorded in four high-power fields for each well, and each experimental condition was assayed in triplicate wells. The number of the cells migrating to the bottom side of the filter was counted and the chemotactic index was calculated as the number of cells migrating toward the test sample divided by the number of cells migrating toward the negative control medium. N-Formyl-Met-Leu-Phe (10^-7 ^M) was used as a positive control. Cyclosporine A (CsA, 10^-6 ^M), anti-CD147 antibody (50 μg/ml) and antagonistic peptide 9 (AP9; 100 nM) were added separately to the upper cells to investigate their effects. The anti-CD147 antibody and antagonistic peptide we used were produced in our laboratory as described previously [16-18].

### Cell culture

The human CD14+ monocytes were isolated from peripheral blood of the patients with RA or from controls using Dynal magnetic human CD14 monocyte isolation kits (Dynal Biotech, Oslo, Norway) according to the manufacturer's directions. The human monocytic THP-1 cells (American Type Culture Collection, Manassas, VA, USA) were cultured in RPMI 1640 medium supplemented with 10% fetal bovine serum (Gibco, Los Angeles, CA, USA), 1% penicillin/streptomycin and 2% L-glutamin at 37°C in a humidified atmosphere of 5% CO_2_. For the induction of cell differentiation, cells (5 × 10^5 ^to 10^6 ^per ml) were seeded in RPMI 1640 medium with 200 nM phorbol myristate acetate (PMA) for 24 h [19]. The human skin fibroblast cells (a kind gift from the Department of Dermatology, Xijing Hospital, Xi'an, China) were cultured in DMEM medium supplemented with 10% fetal bovine serum (Gibco), 1% penicillin/streptomycin and 2% L-glutamin at 37°C in a humidified atmosphere of 5% CO_2_. For the cell co-culture, a fixed number of human CD14+ monocytes/THP-1 cells (1 × 10^4^) or fibroblast cells (1 × 10^4^) were cultured alone or together in serum free DMEM medium supplemented with 1% penicillin/streptomycin and 2% L-glutamin. After a 24 h culture, the supernatant was collected and used for gel zymography.

### Gel zymography

To assess MMP expression in the co-culture of human CD14+ monocytes/THP-1 and fibroblast, media were collected and MMP activity was determined by SDS-PAGE zymography. AP9 (200 μg/ml) was added 24 h in advance to the co-culture of cells to study its effect. Media samples were centrifuged to remove cellular debris, and the supernatant was collected and stored at -20°C. Each sample suspension (30 μl) was mixed with SDS sample buffer without reducing agent and loaded onto a 10% polyacrylamide gel containing 0.1% gelatin. After electrophoresis, gels were washed in 2.5% w/v Triton X-100 and incubated in low salt collagenase buffer containing 50 mmol/l Tris, 0.2 mol/l NaCl, 5 mmol/l anhydrous CaCl_2 _and 0.02% w/v Brij detergent at 37°C for 30 minutes. The gels were subsequently stained with 0.5% Comassie blue (G-250) and were destained with buffer consisting of 20% methanol, 10% acetic acid and 70% distilled water for 30 minutes to visualize the zymogen bands. The zymography gels were scanned and analyzed using US National Institutes of Health Image 1.6 software.

### Invasion assay

The cell invasion assay was performed using 24-well transwell units (Costar, Cambridge, NY, USA) with an 8 μm pore size polycarbonate filter coated with Matrigel (5 μg/ml in cold medium; Becton-Dickinson) to form a continuous thin layer. Prior to the addition of a cell suspension of fixed number (3 × 10^5^), the dried layer of Matrigel matrix was rehydrated with fetal bovine serum free medium (450 μl). AP9 (200 μg/ml) was added in advance to the co-culture of human CD14+ monocytes/THP-1 cells and fibroblasts to study its effect. The cells were then cultured for 24 h at 37°C in a humidified atmosphere of 5% CO_2_. The cells remaining in the upper compartment were completely removed with gentle swabbing. The filter was fixed and stained with haematoxylin and eosin. The cells invading the lower surface of the filter in five microscopic fields of 150 × magnification were counted in each filter. Triplicate samples were acquired and the data were expressed as the average cell number of 15 fields.

### Statistical analysis

Results were expressed as the mean ± SD. The difference between CD147 expression on monocytes in peripheral blood before and after stimulation in the control and RA groups was calculated with a LSD t-test. The difference between CD147 expression on monocytes in the peripheral blood before stimulation in the control and RA groups was calculated with ANOVA. The difference between CD147 expression on monocytes from peripheral blood and synovial fluid in the RA and AS groups was calculated with a Student's t-test. The differences in MMP release and invading cell number in the co-culture of human monocytes/THP-1 cells and fibroblasts in the AP9 and control groups were calculated with Student's t-test. The differences in the chemotactic index between treatment groups and the CyPA control group were calculated with a Dunnett t-test. Spearmen's rho correlation analysis was conducted for the correlation study between CD147 expression and MMP-1 expression, and between CD147 expression and tissue inhibitor of metalloproteinases (TIMP)-1 expression. SPSS software was used for the above analyses and a p-value <0.05 was considered significant.

## Results

### Expression of CD147 on monocytes

#### Comparison between peripheral blood from RA patients and control

**Figure 1:**
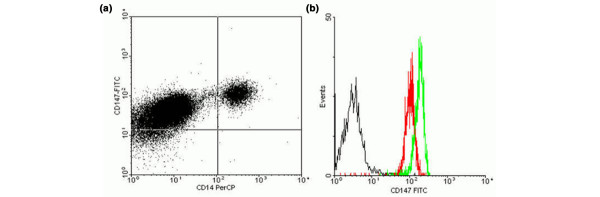
Flow cytometry analysis of CD147 expression on CD14+ cells of peripheral blood. **(a)** CD14 (x-axis) and CD147 (y-axis) staining of leukocytes in peripheral blood of RA patients. In CD14+ cells, the percentage of CD147 positive staining cells was 98.53 ± 2.09%. **(b)** Expression of CD147 on CD14+ cells of peripheral blood in RA and normal control group. The black line shows the isotype control, the red and green lines shows anti-CD147 antibody staining in the normal control and the RA group, respectively. The mean fluorescence intensity (MFI) of CD147 expression on CD14+ cells was higher in the RA than in the normal control group.

The expression of CD147 on CD14+ monocytes was evaluated by flow cytometry. Specifically, two parameters of flow cytometry were used: the mean fluorescence intensity (MFI) and the percentage of positive staining cells. The MFI of CD147 expression on CD14+ monocytes before stimulation was higher in the RA (96.37 ± 14.07) than in the normal control (58.40 ± 8.54) and AS (61.77 ± 15.59) groups, with no significant difference between normal control and AS. It remained almost unchanged after stimulation in the RA group (92.27 ± 22.50) but increased significantly in the normal control group (130.76 ± 17.00, p < 0.05). The percentage of CD147 positive staining cells in CD14+ cells was high in all three groups (normal, 96.82 ± 3.36%; RA, 98.53 ± 2.09%; AS 95.84 ± 3.44%) (Fig. 1a) and no marked change was seen before and after stimulation.

#### Comparison between peripheral blood and synovial fluid from RA patients and control

In the RA group, the MFI of CD147 expression on CD14+ cells of synovial fluid was 131.88 ± 21.04, higher than that in peripheral blood (96.37 ± 14.07, p < 0.01). In the AS group, the MFI of CD147 expression on CD14+ cells of synovial fluid (154.76 ± 27.74) was also higher than that in peripheral blood (61.77 ± 15.59, p < 0.01).

### Expression of CD147, MMP-1 and TIMP-1 on synovium from RA patients and control

**Figure 2:**
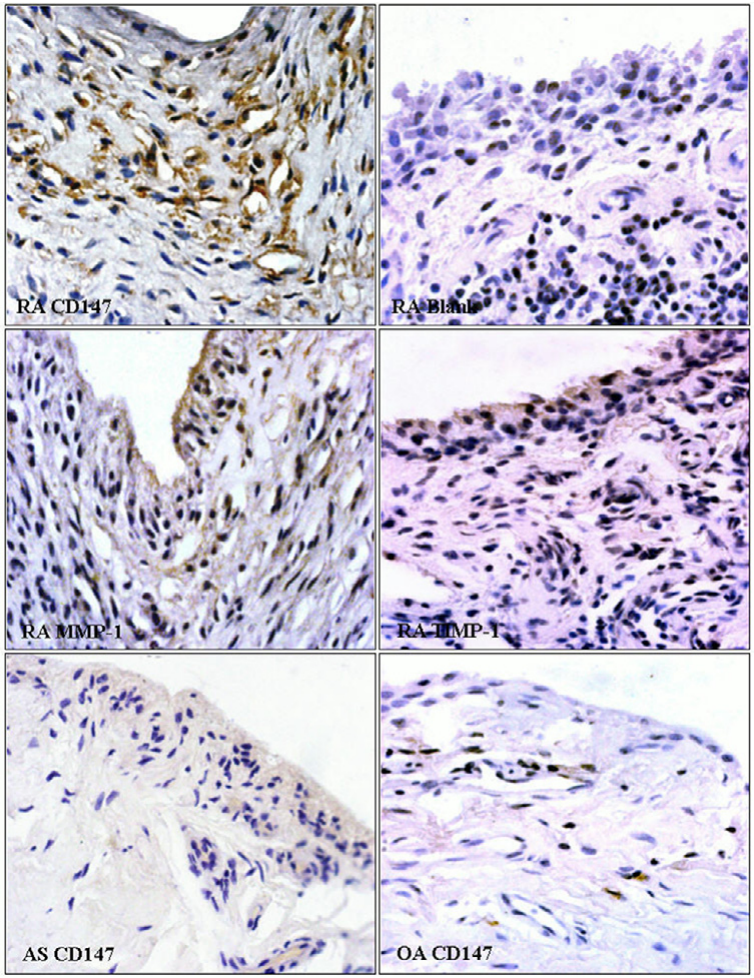
Immunohistochemistry staining of synovium. The top four images show the expression of CD147 (RA CD147), matrix metalloproteinase-1 (RA MMP-1), tissue inhibitor of metalloproteinases-1 (RA TIMP-1) and blank contrast (RA blank) in the lining and sublining layers in RA synovium (× 400). The bottom two images show the expression of CD147 in ankylosing spondylitis (AS CD147) and osteoarthritis (OA CD147) synovium. CD147 was predominantly expressed by macrophage-like cells and fibroblast-like synovial cells in the lining layer and partly by endothelial cells in the rheumatoid arthritis (RA) synovium. The primary antibody used for the blank contrast for streptavidin/peroxides staining was PBS (× 400).

**Figure 3:**
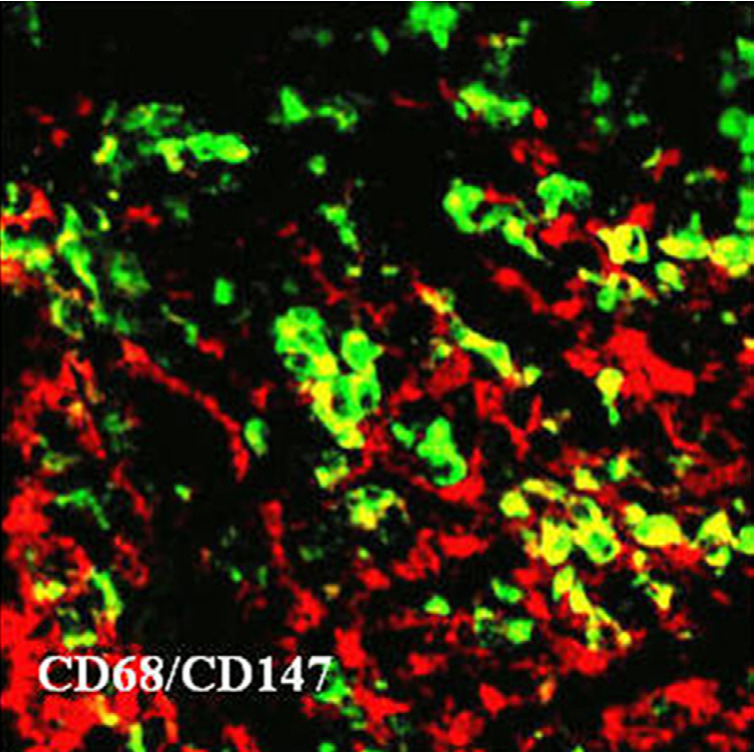
Laser scanning confocal microscope (× 400) image of the double immunofluorescent assay. The expression of CD68+ (green) and CD147+ (red) cells, and the co-expression of CD68+CD147+ cells (yellow) in the lining and sublining layers of rheumatoid arthritis synovium is apparent.

The immunohistochemistry results show that the immunoreactivity of CD147 and MMP-1 was more intense and more widespread in RA synovium than in OA and AS synovium. CD147 was expressed predominantly on the macrophage-like cells and fibroblast-like synovial cells in the lining and sublining layers (Fig. 2). MMP-1 was expressed predominantly on the fibroblast-like synovial cells, macrophage cells and some vascular endothelial cells. Both OA (data not shown) and RA synovium showed positive expression of TIMP-1 in the lining and the sublining layers. The expression of CD147 correlated significantly with that of MMP-1 (p < 0.05), but the expression of CD147 and MMP-1 did not correlate with that of TIMP-1 (p > 0.05) (Table 1). Tissue sections were double immunofluorescent stained and then observed under a LSCM. CD68+/CD147+ cells were observed in the lining layer and the sublining layer of RA synovium (Fig. 3).

### MMP secretion in co-culture of human monocytes/THP-1 cells and fibroblasts

**Figure 4:**
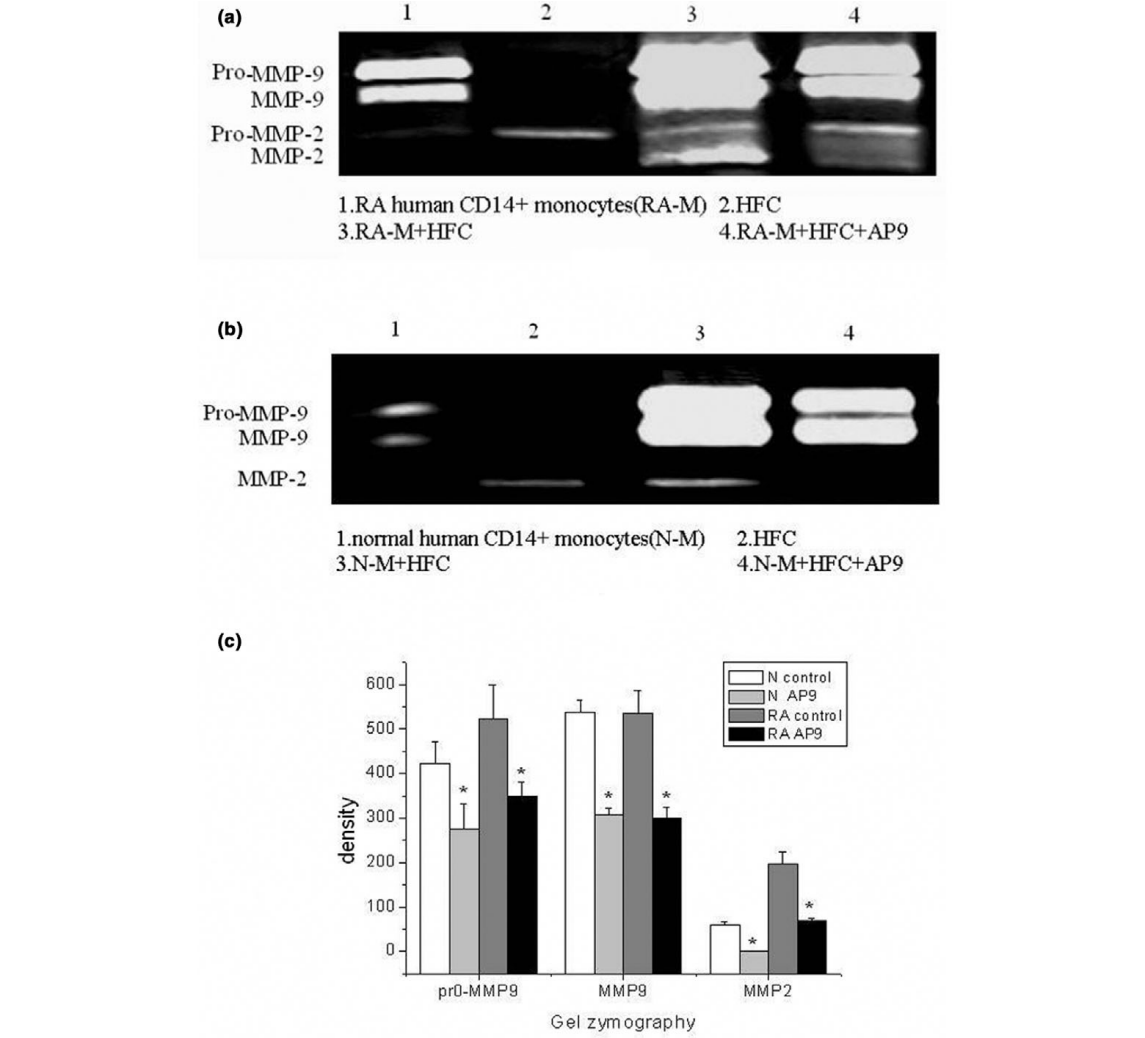
Matrix metalloproteinase (MMP) secretion in a co-culture of CD14+ monocytes and human fibroblast cells (HFC). **(a) **Gel zymography detection of MMP-2 and MMP-9 in human CD14+ monocytes from rheumatoid arthritis (RA) patients, in HFC and in a co-culture of the two. An elevated release and activation of MMP-9 and MMP-2 was seen in the co-culture cells compared with those in the cultures of human monocytes or HFC alone. **(b) **Gel zymography detection of MMP-2 and MMP-9 in human CD14+ monocytes of normal control monocytes, in HFC, and in a co-culture of the two. MMP-2 and MMP-9 release was higher in the co-culture cells. **(c) **Effects of antagonistic peptide 9 (AP9) on pro-MMP-9, MMP-9 and MMP-2 release in a co-culture of human CD14+ monocytes and HFC. The zymography gels above were scanned and the gray density of strips was counted. The release and activation of pro-MMP-9, MMP-9 and MMP-2 was significantly reduced when AP9 was added. Asterisks indicate a p-value <0.01. HFC, Human fibroblast cells.

The gel zymography results show that MMP-9 and MMP-2 secretion increased significantly in the co-culture of isolated human CD14+ monocytes and fibroblasts for both the RA and normal control groups compared with the cultures of human monocytes or fibroblasts alone (Fig. 4). In the RA group, the CD14+ monocytes alone secreted more pro-MMP-9 and MMP-9 than those in the normal control group.

**Figure 5:**
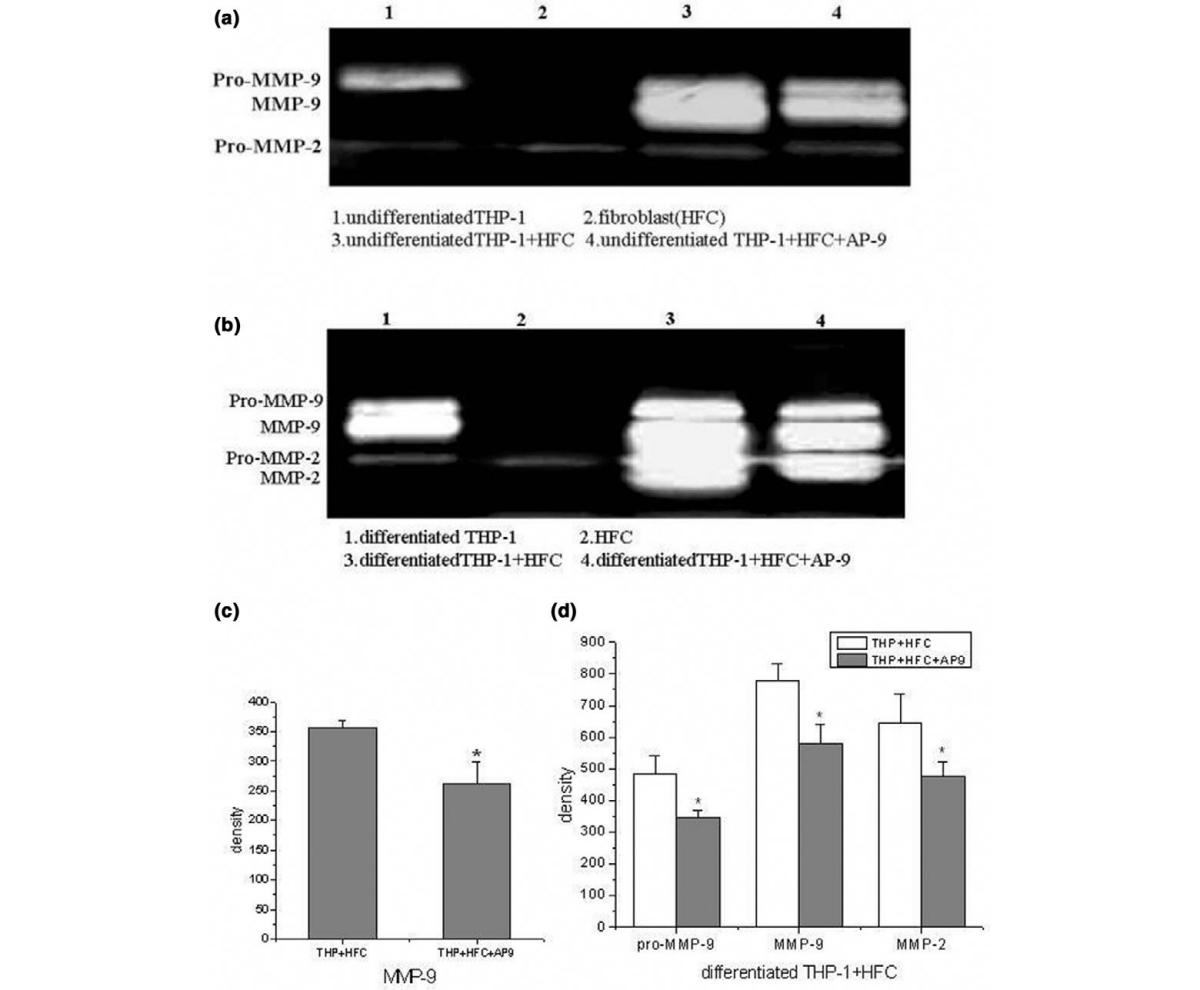
Matrix metalloproteinase (MMP) secretion in a co-culture of THP-1 cells and human fibroblast cells (HFC). **(a) **Gel zymography detection of MMP-2 and MMP-9 in undifferentiated THP-1 cells, in HFC, and in a co-culture of the two. Elevated release and activation of MMP-2 and especially MMP-9 was seen in the co-culture cells compared to the cultures of THP-1 cells or HFC alone. **(b) **Gel zymography detection of MMP-2 and MMP-9 in phorbol myristate acetate (PMA)-induced differentiated THP-1 cells, in HFC, and in a co-culture of the two. Pro-MMP-2 and MMP-2 release was higher in the co-cultured cells. PMA-induced differentiated THP-1 cells secreted more pro-MMP-9 and MMP-9 compared with undifferentiated THP-1 cells. **(c) **Effects of CD147 antagonistic peptide 9 (AP9) on MMP-9 release in a co-culture of undifferentiated THP-1 cells and HFC. The zymography gels above were scanned and the gray density of strips was counted. The release and activation of MMP-9 was significantly reduced when AP9 was added. The asterisk indicates a p-value < 0.01. **(d) **Effects of AP9 on the release of pro-MMP-9, MMP-9 and MMP-2 in a co-culture of differentiated THP-1 cells and HFC. Pro-MMP-9, MMP-9 and MMP-2 secretion all decreased significantly in the AP9 group. Asterisks indicate a p-value < 0.01. HFC, Human fibroblast cells.

To verify the above findings, we also used a PMA-induced cell differentiation model of the human monocytic cell line THP-1 cells. THP-1 cells were used because of their high expression of CD147 shown in previous reports [7] and our own experiments (data not shown). The co-culture of undifferentiated THP-1 cells and fibroblasts showed a significantly elevated level of release and activation of MMP-2 and particularly MMP-9 compared with the cultures of THP-1 cells or fibroblasts alone (Fig. 5a). PMA-induced differentiated THP-1 cells also secreted more pro-MMP-9 and MMP-9 compared with undifferentiated THP-1 cells (Fig. 5b). Elevated secretion of pro-MMP-2 and MMP-2 was observed in the co-culture of differentiated THP-1 cells and fibroblasts (Fig. 5b).

### Invasive ability of cells in co-culture of human monocytes/THP-1 cells and fibroblasts

The functional cell invasion assay showed that the invasive ability of human CD14+ monocytes in the RA group and normal control group were different. The number of human CD14+ monocytes that invaded through the filter in the RA group (858.3 ± 57.5) was higher than that in the normal control group (602.3 ± 126.7, p < 0.05). In the co-culture of human monocytes and fibroblasts, the cell number in the RA group (1235.7 ± 137.6) was also higher than that in the normal control group (918.3 ± 117.5, p < 0.05).

**Figure 6:**
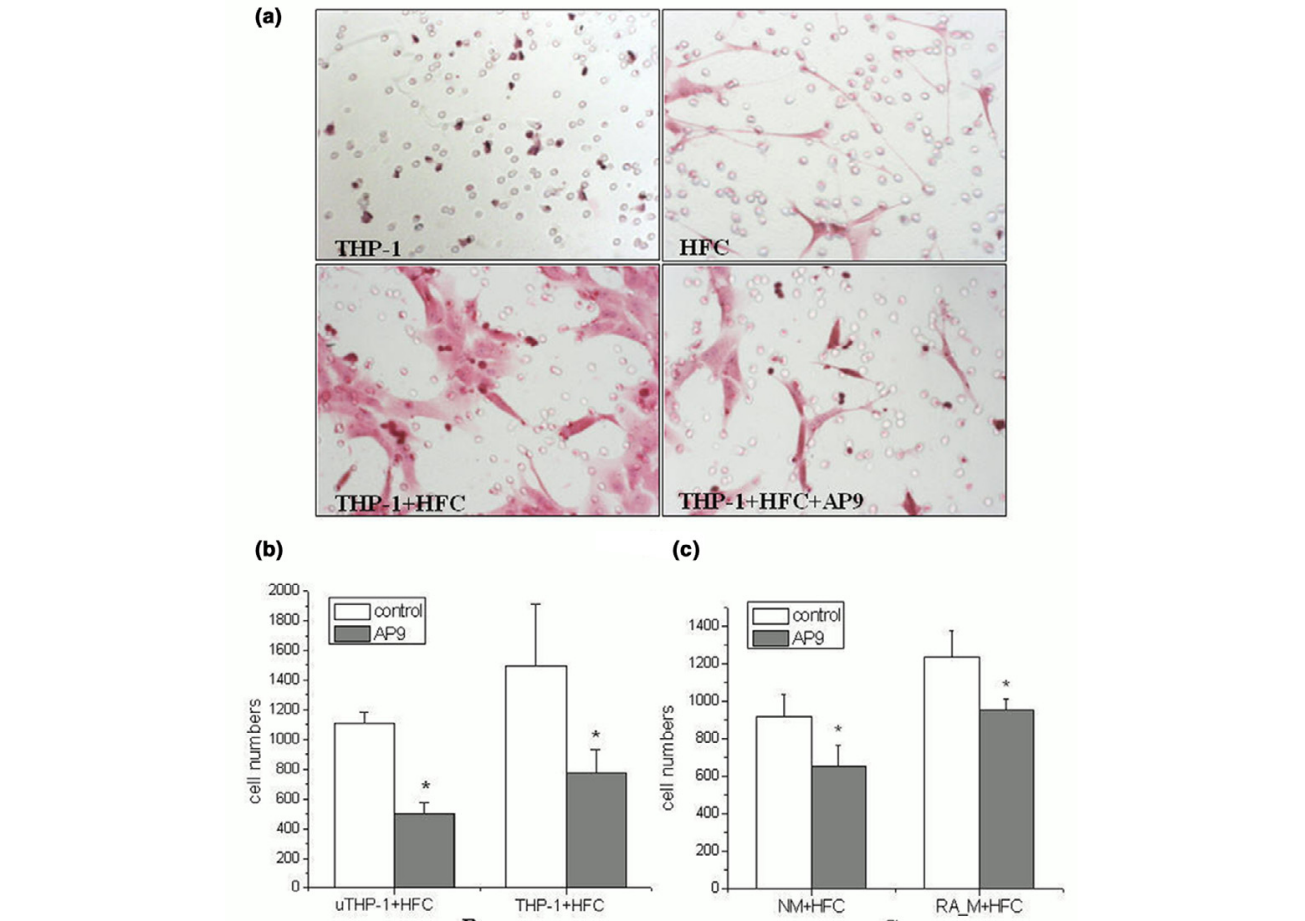
Effect of CD147 antagonistic peptide 9 (AP9) on the invasive ability of the co-culture cells. **(a) **The haematoxylin and eosin staining results of the lower surface filters show that the cells had invaded through the filter and attached to the lower side of the filter (× 150). In the co-culture of undifferentiated THP-1 cells and HFC, the number of cells was increased compared with the cultures of THP-1 cells or HFC alone. **(b) **Effect of AP9 on the number of cells that invaded through the Matrigel coated filter in the co-culture of undifferentiated or differentiated THP-1 cells and HFC. The number of cells that invaded through the filter was counted, showing that when AP9 was added the number of cells decreased. Asterisks indicate a p-value <0.01. **(c) **Effect of AP9 on the number of cells that invaded through the Matrigel coated filter in a co-culture of human CD14+ monocytes and HFC. The number of cells decreased when AP9 was added. Asterisks indicate a p-value < 0.05. HFC, Human fibroblast cells.

The pictures of filters show that the number of cells that invaded through the filter in the co-culture of undifferentiated THP-1 cells and fibroblasts was significantly increased compared with the cultures of THP-1 cells or fibroblasts alone (p < 0.05) (Fig. 6a). When the THP-1 cells were induced to differentiate, the number of invading cells (865.7 ± 113.9) was higher than that of undifferentiated THP-1 cells (478 ± 70.1, p < 0.05), and the number of cells that invaded in the co-culture of differentiated THP-1 cells and fibroblasts (1493.7 ± 417.5) was also higher than that in the co-culture of undifferentiated THP-1 cells and fibroblasts (1108.3 ± 73.4, p < 0.05).

### Effect of AP9 on MMP release, activation and the invasive ability of cells in co-culture of human monocytes/THP-1 cells and fibroblasts

To identify the relationship between CD147 and the production of MMPs, AP9 was added into the co-culture system of human monocytes/THP-1 cells and fibroblasts and its effect on MMP activity and the invasive ability of these cells was observed. AP9 had some inhibitory effect on the secretion of pro-MMP9 (control, 33.7 ± 18.5%; RA, 31.8 ± 16.2), MMP-9 (control, 41.9 ± 5.8%; RA, 43.8 ± 5.1%) and MMP-2 (control, 100%; RA, 63.9 ± 6.2%) in the co-culture of human monocytes and fibroblasts in both the normal control and RA groups (Fig. 4c).

A significantly reduced release (26.5 ± 11.7%) of MMP-9 in the co-culture of undifferentiated THP-1 cells and fibroblasts was observed in the AP9 group compared with that without AP9 (p < 0.01; Fig. 5c). The pro-MMP-9, MMP-9 and MMP-2 secretion in the co-culture of differentiated THP-1 cells and fibroblasts decreased significantly (27.5 ± 12.7%, 25.4 ± 12.4% and 25.5 ± 3.8%, respectively) in the AP9 group compared with those without AP9 (p < 0.01; Fig. 5d).

In the cell invasion assays, the number of cells that invaded through the Matrigel coated filter decreased following a 24 h treatment with AP9 (200 μg/ml) in the co-culture cells (Fig. 6b, c). AP9 inhibited cell invasion in the co-culture of human monocytes and fibroblasts by 28.9 ± 5.9% in the normal control group and by 22.8 ± 3.8% in the RA group. AP9 inhibited cell invasion by 55.1 ± 4.5% in the co-culture of undifferentiated THP-1 cells and fibroblasts and by 44.1 ± 22.9% in the co-culture of differentiated THP-1 cells and fibroblasts.

### Chemoattraction of CyPA for peripheral mononuclear cells in RA and its blockage by anti-CD147 antibody and AP9

**Figure 7:**
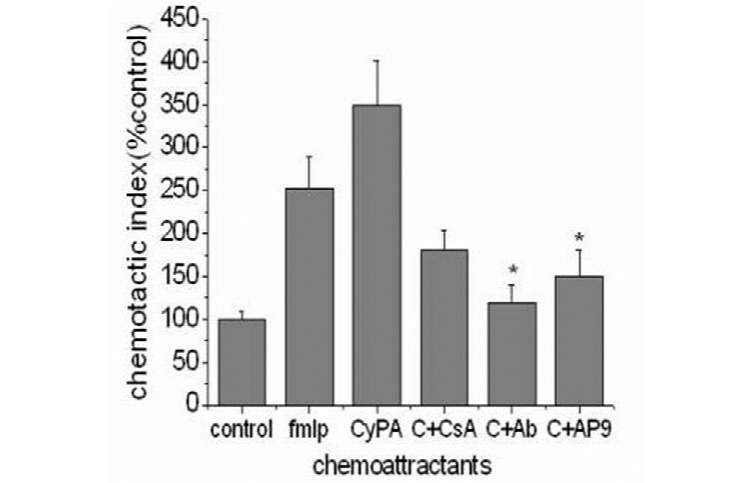
Effect of anti-CD147 antibody (Ab) and antagonistic peptide 9 (AP9) on the chemoattraction of cyclophilin A (CyPA). The chemotactic indexes decreased significantly when anti-CD147 antibody or AP9 was added. CsA, cyclosporine A. C, CyPA. fmlp, n-Formyl-Met-Leu-Phe, positive control.

Based on the reports that CD147 is a high affinity receptor for CyPA and responsible for a cyclophilin signaling cascade that culminates in extracellular signal-regulated kinase (ERK) activation and chemotaxis [20,21], we examined the chemoattraction of CyPA for peripheral mononuclear cells in RA and the blockage effect of anti-CD147 antibody and AP9 on this. The optimum chemotaxis dose of CyPA was found to be 100 ng/ml. The CyPA chemotactic index for peripheral mononuclear cells in RA patients (350 ± 52% control) was higher than that in the normal control group (252 ± 63% control, p < 0.05), indicating that CyPA had some significant chemotactic effect on these cells (p < 0.05). The chemotactic indexes decreased significantly when anti-CD147 antibody or AP9 was added to the mononuclear cells in RA (120 ± 27% and 150 ± 40% control, respectively; p < 0.01) (Fig. 7). The blockage of CyPA chemoattraction for the mononuclear cells by anti-CD147 antibody and AP9 was more obvious than that caused by CsA (p < 0.05).

## Discussion

Our results on the expression of CD147 on peripheral blood monocytes in normal subjects are consistent with previous studies demonstrating that CD147 is expressed on the surface of activated monocytes [7,22-24]. The expression of CD147 on normal inflammatory cells and many normal tissues [25-27] is suggestive of its physiological role in some situations, perhaps in which increased MMP expression is involved in tissue remodeling. In RA patients, however, we found that the expression density of CD147 on monocytes from peripheral blood, as well as those from synovial fluid, was much higher than normal. Following stimulation with IFN-γ, the expression of CD147 on monocytes from normal subjects was upregulated. But *in vitro *IFN-γ stimulation seemed to have little effect on peripheral monocytes from RA patients, possibly because of the already activated condition of RA monocytes *in vivo*. These results indicate that the upregulation of CD147 expression on monocytes is possibly associated with the pathogenesis of RA and CD147 may play an important role in the cartilage and bone destruction of RA.

To confirm the expression of CD147 in synovium and to characterize the CD147 expressing cells, immunohistochemical staining and immunofluorescence were performed. The immunoreactivity of CD147 was more intense and more widespread in RA synovium than in OA and AS synovium, and the expression of CD147 correlated with MMP-1 expression. These results are in part consistent with some previous reports [12,13], but our immunohistochemical staining and immunofluorescence results further confirmed that CD147 is expressed in synovium not only on the fibroblast-like cells and granulocytes, but also on the CD68+ macrophage-like cells, which are believed to be peripheral monocyte-derived. As the expression of CD147 is high in peripheral blood monocytes in RA patients, it is highly possible that this will be maintained and will stimulate MMP production after the monocytes infiltrate into joints and differentiate into macrophage-like synoviocytes. The findings of Major *et al*. [28] that CD147 expressed on differentiated monocytes and CD68-positive macrophages in human atherosclerotic plaques also support our findings.

The upregulation of CD147 expression on monocytes/macrophages suggests that CD147 may be important in both the autocrine and paracrine stimulation of MMP expression. It has been shown that homophilic CD147-binding occurs in the context of both heterotypic and homotypic cell-cell interactions and that CD147 can itself be a receptor to induce MMP production not only in primary fibroblast cells but also in tumor cells [10]. On the basis of this, we presume that the increased expression of CD147 on macrophages in RA synovium could possibly induce MMP production through interaction with surrounding fibroblast-like synoviocyts and also with other macrophages. Our *in vitro *studies show that a co-culture of human CD14+ monocytes/THP-1 cells and human fibroblasts resulted in higher levels of MMP-2 and MMP-9 in culture supernatants compared to human monocytes or fibroblasts alone. This fibroblast triggered enhanced production could be suppressed, however, by a peptide antagonistic to CD147/HAb18G produced as described before [16-18]. (HAb18G is abundantly expressed in human hepatoma tissues and on the cell surface of several hepatoma cell lines with a highly metastatic potential [18] and is a new member of CD147 family [6,29].) The results of our cell invasion assay confirmed the results of our gel zymography assay, which partly prove that CD147 upregulation on macrophages may increase MMP production through both autocrine and paracrine stimulation and that macrophages may act as an amplifier of the pathogenetic cascade in RA via an increase in MMP production by interacting macrophages and fibroblasts.

Bukrinsky and colleagues have reported that CD147 is a high affinity receptor for CyPA and have demonstrated that it is responsible for a cyclophilin signaling cascade that culminates in ERK activation and chemotaxis [20,21]. It is also found that CyPA, which belongs to the immunophilin family of peptidyl-prolyl cis-trans isomerases [30-32], have chemotactic activity towards eosinophils or neutrophils [33] and accumulate in synovial fluids of patients with RA [34]. If CyPA is released into the medium during inflammation, it is highly possible that it could act as a ligand of CD147 to induce the accumulation of inflammatory cells that highly express CD147 in the joints of RA patients. The results of our *in vitro *chemotaxis assays confirm that CyPA has a chemotactic effect on peripheral blood mononuclear cells. Moreover, we have also found that this chemotaxis can be significantly suppressed by adding an anti-CD147 monoclonal antibody or antagonists of CD147. This suggests that CyPA does interact with CD147, although the actual role of CyPA in CD147 function and in RA still needs to be elucidated.

## Conclusion

We conclude in this study that the increased expression of CD147 on monocytes/macrophages in RA may be responsible for elevated MMP secretion, cell invasion and the CyPA-mediated cell migration into joints, all of which may contribute to the cartilage and bone destruction of RA. These findings, together with a better understanding of the relationship between CD147, CyPA and RA and of the possible mechanism and regulation of the effect of CD147 on MMP production, will help in the development of innovative therapeutic interventions for RA.

## Abbreviations

AP = antagonistic peptide; AS = ankylosing spondylitis; BSA = bovine serum albumin; CsA = cyclosporine A; CyPA = cyclophilin A; EMMPRIN = extracellular matrix metalloproteinase inducer; ERK = extracellular signal-regulated kinase; IFN = interferon; IL = interleukin; LSCM = laser scanning confocal microscope; MFI = mean fluorescence intensity; MMP = matrix metalloproteinase; OA = osteoarthritis; PBS = phosphate buffered saline; PMA = phorbol myristate acetate; RA = rheumatoid arthritis; SP = streptavidin/peroxides; TIMP-1 = tissue inhibitors of metalloproteinases; TNF = tumor necrosis factor.

## Competing interests

The authors declare that they have no competing interests.

## Authors' contributions

PZ participated in the design of the study and drafted the manuscript. JD carried out the flow cytometry assay and the chemotaxis assay, performed the statistical analysis and helped to draft the manuscript, and is one of the co-first authors. JZ performed the invasion and gel zymography assays, and is one of the co-first authors. WD participated in the immunohistochemistry staining and immunofluorescent assay, and is one of the co-first authors. CF carried out the flow cytometry assays. ZC participated in the design of the study and helped to draft the manuscript, and is the corresponding author. All authors read and approved the final manuscript.

## Figures and Tables

**Table 1:** Results of immunohistochemistry staining of the synovium of 12 rheumatoid arthritis patients

	1	2	3	4	5	6	7	8	9	10	11	12
CD147	++	++	++	++	+	+	++	+	+	++	+	++
MMP-1	++	+	+	++	+	+	++	+	+	++	+	++
TIMP-1	+	+	+	+	-	+	+	+	+	+	+	-

Positive intensity is indicated as: -, positive cell ratio <5%; +, ratio 5% to 50%; ++, ratio >50%. Spearmen's rho analysis showed that expression of CD147 correlated significantly with matrix metalloproteinase-1 (MMP-1) expression (p < 0.05), but that the expression of CD147 and MMP-1 did not correlate with tissue inhibitors of metalloproteinases-1 (TIMP-1) expression (p > 0.05).
